# Conceptualising learning healthcare systems and organisations in the context of rehabilitation: a scoping review protocol

**DOI:** 10.12688/hrbopenres.13614.1

**Published:** 2022-10-11

**Authors:** Lauren Christophers, Zsofia Torok, Catherine Cornall, Aoife Henn, Clare Hudson, Teresa Whyte, Diarmuid Stokes, Aine Carroll

**Affiliations:** 1 School of Medicine, University College Dublin, Dublin, Ireland; 2 National Rehabilitation Hospital, Dublin, Ireland; 3 UCD Library, University College Dublin, Dublin, Ireland

**Keywords:** Learning healthcare system, learning organisation, rehabilitation, systems thinking, complexity

## Abstract

**Background:** Transformative system wide action is needed for healthcare systems to meet the needs of an increasing aging population and changing health needs. One idea is that health systems can become “learning organisations” (LO) or “learning healthcare systems” (LHS) that continuously generate and apply evidence, innovation, quality, and value to provide better care. This is of value to non-acute healthcare settings such as rehabilitation, which are complex, multi-dimensional and multi-disciplinary in nature. Little is known about how these frameworks have been applied to rehabilitation settings.

**Objective and inclusion criteria:** The aim of this scoping review is to systematically map and summarise the literature conceptualising and operationalising LHS and LO in rehabilitation settings.

Studies will be included which define a LO or LHS; or describe an operating LHS/LO; or include the translation of research evidence generated from LHS/LO data into healthcare improvement within a rehabilitation context will be included. Study designs such as quantitative, qualitative, mixed method studies, and case studies will be included.

**Methods:** The guidelines from the Joanna Briggs institute methodology for scoping reviews will be used for this review. The literature search will be performed using a three-step search strategy: an initial limited search of two databases has been performed to identify relevant key words and index terms. The developed search string will be adapted and applied across the following databases: OVID MEDLINE, EMBASE, CINAHL Plus, APA PsycINFO and COCHRANE Database of Systematic Reviews. This will be followed by search of the reference lists of selected sources and relevant data-hubs. A draft data extraction framework will be used and updated iteratively to extract data. Frequency counts and qualitative content analysis will be employed to address the research question of how LHS and LO have been conceptualised and operationalised in the context of rehabilitation.

## Introduction

With an increasing aging population, changing health needs and growing public expectations for new ambitious health goals, the pressure is on for health systems to produce better health outcomes. However, as highlighted by Kruk *et al.* (2018) - transformative, system wide actions are needed globally, to meet such goals. An upheaval of this nature to healthcare systems is majorly challenging, and particularly so in current times when healthcare organisations globally are struggling with fragmentation between services and system inefficiencies. Major changes in how health care is designed and delivered will clearly be needed to meet the challenges of ageing populations, chronic diseases and health inequities ( Kruk *et al.*, 2018). Integrated care has been identified as a helpful strategy, where health and social services are delivered through comprehensive care pathways organised around patient needs ( Robertson, 2011). Within models of integrated care there should be horizontal co-ordination, which spans disciplinary and professional boundaries as well as vertical co-ordination, with a continuum from primary to secondary to tertiary care. In such models, care should not be episodic in nature and an effort should be made to minimise unplanned stays in acute care. The World Health Organization (WHO) has emphasised the value of integrated, person centred care, as building blocks for universal health coverage and higher quality of care ( World Health Assembly, 2016). However, questions remain for health systems researchers around the implementation of integrated care. How can we design solutions for healthcare which will best facilitate the major global shift required to meet complex challenges facing healthcare providers today? 

A potential transformation to healthcare systems that has gained traction over recent years is the idea that healthcare systems can become "learning" systems/organisations. This aligns well with the WHO definition of integrated care, which emphasises the importance of feedback loops to continuously improve performance as well as multi-disciplinary teams of providers ( World Health Assembly, 2016). The literature on this topic includes a diverse range of terminology from across different disciplines (such as implementation science, organisational development, and healthcare management) that we will endeavour to explicate below.

The concept of a learning organisation (LO) originated in the 1990s with the seminal work of Peter Senge and others, who outlined how to build adaptive organisations which learn and lead change. This early work was focused on developing frameworks and tools to operationalise this concept within actual organisations ( Garvin, 2000; Marquardt, 1996; Senge, 2006). Much of this work was grounded in exploration and analysis of private companies – with growing recognition of the competitive advantage of investing in knowledge and learning. Over time, the LO concept has also been applied to public organisations. However, researchers have recognised the need for LO frameworks to be sufficiently complex and dynamic to truly accommodate public organisations ( Rashman *et al.*, 2009). Frameworks for a LO which are grounded in Senge’s original conception can meet this need. The five pillars of learning organisations as he outlined them were personal mastery, mental models, building a shared vision, team learning and "the fifth discipline" - systems thinking. Systems thinking involves an appreciation of the complexity of interacting components of an organisation. Thus, the fifth discipline also integrates the other four ( Iles & Sutherland, 2001) and champions a complexity lens. A complexity approach is particularly essential in the examination of healthcare systems and delivery.

Healthcare systems are characterised by being complex. This complexity emerges from multiple independent agents interconnected in a surrounding environment, with continuous evolution and adaptation – thus healthcare systems qualify as complex adaptive systems ( Carroll, 2021; Peters, 2014). Because of this, systems thinking has been identified as an appropriate tool in thinking about complexity in healthcare ( Rusoja *et al.*, 2018). Complexity thinking has been increasingly embraced in healthcare with an understanding that more traditional industrial oriented healthcare solutions can be reductionist ( Waldman, 2007), and that problems in healthcare should be viewed through a complexity lens with complexity sensitive solutions ( Khan *et al.*, 2018; Plsek & Greenhalgh, 2001). Healthcare researchers have noted that this thinking aligns well with popular perspectives and philosophies recently promoted in healthcare – e.g., collectivist leadership, person centredness and interventions in healthcare which require new behaviours from those delivering or receiving the intervention ( Carroll, 2021; Khan *et al.*, 2018). Systems thinking can support change within health service delivery ( Sarkies *et al.*, 2021), through effective conceptualisation of problems and context in healthcare allowing effective work with stakeholders ( Haynes *et al.*, 2020) thus providing insights to improve healthcare quality.

 Thus, systems thinking is useful in healthcare innovation as it incorporates a complexity lens and complexity sensitive solutions to problems in healthcare. However, common criticisms of “learning organisations” as a concept are that there are contradictory definitions and vague concepts ( Shea & Taylor, 2017). Some scholars refer to this concept as an outcome while others conceptualise it as a process “organisational learning” ( Argyris & Schön, 1978; Bateson, 1979). A scoping review of the application of LO to healthcare systems ( Akhnif *et al.*, 2017) noted the versatility of the application of the concept – that it has been applied to different levels of healthcare systems, in descriptive, prescriptive, and analytical manners. Alongside diversity in the application of the concept, there was also consensus across papers about the transformative potential of the LO concept for promoting learning and healthcare reformation. This demonstrates another longstanding critique of LO - despite the potential of the concept, there is a lack of empirical evidence on how to effectively apply LO frameworks and support organisational learning in practice ( Huber, 1991; Tosey *et al.*, 2012; Visser, 2007). Further research is required to build rigorous evidence on the impact of applying LO frameworks to healthcare organisations. This review will contribute to knowledge of how healthcare organisations can become LOs, while focused on a specific context – that of rehabilitation care and rehabilitation organisations. The focus on this context may eliminate some of the variation and inconsistencies between different applications of the LO concept – as Akhnif *et al.* noted that researchers needed to adapt existing frameworks for their context.

Within healthcare literature, a similar (though potentially separate) concept to the LO has emerged and greatly grown in popularity – that is the "learning healthcare system" (LHS). As with the LO concept, the core idea is for health systems and organisations to continuously learn and adapt. However, definitions of LHS revolve around a data driven approach to healthcare, in which data routinely collected as a part of care are used in healthcare improvement. For example: “ *We define a LHS as a system in which routine health practice data, from service delivery and patient care, can lead to iterative cycles of knowledge generation and healthcare improvement*" ( Enticott *et al.*, 2021, p. 2). Commonly cited definitions have arisen from the Institute of Medicine in the US - “ *science, informatics, incentives, and culture are aligned for continuous improvement and innovation, with best practices seamlessly embedded in the delivery process and new knowledge captured as an integral by-product of the delivery experience*” ( Institute of Medicine, 2011; Institute of Medicine, 2014). Similar problems persist as those from the field of organisational learning with varying definitions and inconsistent use of terminology (as evident in our table of search terms, see Table 1.). However, commonalities and core features of the concept have been identified. Defining features include cyclical data driven approaches, embedded researchers and academic partnerships and an emphasis on being service led and community led ( Enticott *et al.*, 2021; Johnson *et al.*, 2021). Co-design with knowledge stakeholders has been highlighted as a key part of successful implementation of any "learning" model of healthcare ( Johnson *et al.*, 2021).

**Table 1.** Keywords for the search strings.

Learning health system	Rehabilitation	Exclude
learning health*	rehabilitati*	addict*
LHS	physiotherapy	alcohol *
rapid-learning	physical therapy	substance
RLHS	occupational therapy	abuse
learning organisation	neurorehabilitation	misuse
systems thinking	habilitation	mental health
		cardiac
		pulmonary
		narcotic*
		psychiatry*

Barriers to the successful implementation of these models largely relate to financial obstacles and infrastructural impediments. Researchers have noted difficulties in obtaining external funding as agencies which support clinical research have historically valued randomised controlled trials over robust observational study designs best suited to LHS research ( Bindman *et al.*, 2018; Forrest *et al.*, 2018). With data driven approaches that have gained popularity, particularly in the US, the success is dependent on the existence of electronic health records and infrastructure. Gaps in the academic literature continue despite the recent popularity of the concept, with a significant gap around *how* to create and sustain a LHS ( Budrionis & Bellika, 2016). Previous evidence syntheses have been conducted on this topic ( Budrionis & Bellika, 2016; Enticott *et al.*, 2021; McLachlan *et al.*, 2018; Platt *et al.*, 2020; Pomare *et al.*, 2022). However, there has been no review published focused on learning health systems within the specific context of rehabilitation. LHS is a concept which has been used in a wide variety of settings including oncology, surgery, cardiology, primary care and paediatrics ( Platt *et al.*, 2020), but far more rarely in rehabilitation setting.

The concepts of LHS and LO have great potential utility for rehabilitation organisations. As healthcare organisations and systems move towards electronic health records, generating data from existing practices, creating knowledge, and feeding this knowledge back into practice could be highly beneficial to rehabilitation practice. A recent systematic review reported benefits across settings and countries which included benefits to patients such as self-management opportunities and evidence-based care, benefits to system level performance such as identification of patients for follow up, specialised care and longitudinal tracking and benefits to research through enablement of real-world research and evaluation ( Enticott *et al.*, 2021). Furthermore, many of Senge's pillars of a learning organisation are highly relevant to rehabilitation such as team learning and building a shared vision. This is evident from definitions of rehabilitation for example:

“ *Rehabilitation is a process of active change by which a person who has become disabled acquires the knowledge and skills needed for optimal physical, psychological and social function*”

( *National Policy/Strategy for the Provision of Rehabilitation Services*, 2009, p. 3) - based on the definition by the British Society of Rehabilitation Medicine ( Turner-Stokes & Wade, 2004)

Such definitions illustrate the multi-dimensional, multi-disciplinary nature of rehabilitation care which evidently requires an expert, skilled interdisciplinary team who work towards patient-centred goals over time and across settings as the patient changes ( Wade, 2015). Thus, we propose that the concepts relevant to becoming a learning organisation or healthcare system have the potential to offer useful frameworks for rehabilitation that align with the priorities of integrated services and person-centred care. With the shift to electronic health records, now is the time to explore and test such possibilities. The first step in this regard must be in clarifying terminology, solidifying a clear conceptualisation of what is meant by "learning" organisations and healthcare systems. This work should be explored and applied to different healthcare settings - including rehabilitation and other non-acute settings.

This review will offer a unique contribution to healthcare improvement literature, as the first evidence synthesis of the concept of a "learning" healthcare system or organisation in the context of rehabilitation care. A scoping review has been identified as the appropriate method of evidence synthesis for this topic. The Joanna Briggs Institute evidence synthesis manual defines scoping reviews as a type of evidence synthesis which allows researchers to systematically map the breadth of evidence available on a topic, identify key features of a concept and clarify definitions of a concept ( Munn *et al.*, 2022). In this case, a scoping review will allow insight into a) the volume of papers on these topics of LO and LHS within rehabilitation literature b) the varying definitions and terminology, which has previously been identified as a problem with these concepts c) the similarities and differences between the two concepts and d) the key features of these concepts as they apply to rehabilitation.

### Review question

How have “learning health systems” and “learning organisations” been conceptualised and operationalised in the field of rehabilitation?******

The objectives of this review are:

•     To gather and note the prevalence of literature which applies LHS and LO concepts to the rehabilitation context.

  •   From this literature, to collate and synthesise different definitions of learning healthcare organisations and systems.

  •   To record how LO or LHS frameworks were applied in rehabilitation settings and the changes made within the organisation.

  •   To note the impact of the application of these frameworks and changes made within the organisation.

## Methods

### Screening protocol

The screening protocol is based on the guidelines from the Joanna Briggs Institute (JBI) Manual for Evidence Synthesis and Levac ( Levac *et al.*, 2010; Peters *et al.*, 2020). At the beginning of the process, the team will meet to discuss decisions surrounding study inclusion and exclusion. Two reviewers will dual screen all abstracts and a third reviewer will resolve any conflict and decide on final inclusion.

### Electronic databases

The research team will undertake a comprehensive search of the literature within the following databases:

OVID MEDLINE

EMBASE

CINAHL Nursing and Allied Health (CINAHL Plus)

APA PsycINFO

COCHRANE Database of Systematic Reviews

### Key search terms and search strings

The key search concepts for this study are ‘learning organisation’ AND ‘rehabilitation’. Alternative and related terms for each of the concepts will also be included, and exclusion terms will be added to eliminate results related to substance abuse and acute care rehabilitation. Table 1 contains the keywords and exclusion terms for the search strings. The search query will be adapted for each database using specific Boolean operators, truncation markers, and MeSH-, Emtree-, subject- and index terms and headings where necessary. The research team collaborated with an expert university librarian (D.S.) in designing and refining the search strategy. Table 2 contains the search strings for each database.

**Table 2.** Search strings for each database.

Database	Core search (title, abstract)	MeSH-, Emtree-, subject- or index terms
OVID MEDLINE	"Learning health*" or LHS or Rapid-learning or RLHS or "Learning organisation" or "systems thinking" and Rehabilitati* or physiotherapy or "physical therapy" or "occupational therapy" or neurorehabilitation or habilitation	exp Learning Health System/ or *Organizational Innovation/ and *Rehabilitation/ or *Hospitals, Rehabilitation/ or *"Physical and Rehabilitation Medicine"/ or *Stroke Rehabilitation/ or *Neurological Rehabilitation/ or *Rehabilitation Centers/ or *Rehabilitation Research/
EMBASE	'learning health*' OR lhs OR 'rapid learning' OR rlhs OR 'learning organisation' OR 'systems thinking' AND rehabilitati* OR physiotherapy OR 'physical therapy' OR 'occupational therapy' OR neurorehabilitation OR habilitation	'learning health system'/exp AND 'stroke rehabilitation'/mj OR 'occupational therapy'/mj OR 'sensorimotor integration'/mj OR 'speech and language rehabilitation'/mj OR 'neurorehabilitation'/mj
CINAHL	"Learning health*" OR LHS OR Rapid-learning OR RLHS Or "Learning organisation" OR "Learning Based" OR "systems thinking" AND Rehabilitati* OR physiotherapy OR “physical therapy” OR “occupational therapy” OR neurorehabilitation OR habilitation	MM "Learning Health System" AND MM "Activities of Daily Living+") OR (MM "Rehabilitation") OR (MM "Occupational Therapy") OR (MM "Physical Therapy"
PsycINFO	“Learning health*” OR Rapid-learning OR RLHS OR “Learning organisation” OR “systems thinking” AND Rehabilitati* OR physiotherapy OR "physical therapy" OR "occupational therapy" OR neurorehabilitation OR habilitation	subject("Organizational Learning ") AND MJMAINSUBJECT.EXACT("Rehabilitation") OR MJMAINSUBJECT. EXACT("Rehabilitation Centers") OR MJMAINSUBJECT.EXACT ("Neurorehabilitation") OR MJMAINSUBJECT.EXACT("Vocational Rehabilitation") OR MJMAINSUBJECT.EXACT("Telerehabilitation")
COCHRANE	"Learning health*" OR LHS OR Rapid-learning OR RLHS Or "Learning organisation" OR "Learning Based" OR "systems thinking" AND Rehabilitati* OR physiotherapy OR “physical therapy” OR “occupational therapy” OR neurorehabilitation OR habilitation	[Learning Health System] OR [Organizational Innovation] AND [Rehabilitation] OR [Rehabilitation Centers] OR [Hospitals, Rehabilitation] OR [Physical and Rehabilitation Medicine] OR [Rehabilitation of Speech and Language Disorders] OR [Rehabilitation Research] OR [Neurological Rehabilitation] OR [Telerehabilitation] OR [Stroke Rehabilitation]

As recommended by the JBI Manual for Evidence Synthesis ( Peters *et al.*, 2020), a three-step process for applying a search strategy will be implemented. Step 1 has already been carried out and involved an initial limited search on multiple databases for relevant articles. This has been followed by analysis of the keywords and phrases contained in the titles and abstracts of the retrieved papers, and of the index terms used to describe the articles. Step 2 will involve the search across all included databases using the identified key words outlined in Table 1. Step 3 will involve the search of the reference lists of selected sources to identify any additional relevant studies, as well as hand search of relevant data-hubs.

### Inclusion/exclusion criteria

In line with methodological framework by Arksey & O’Malley (2005), the final inclusion and exclusion criteria will be devised post hoc, based on increasing familiarity with the literature. Following the JBI guidelines, however, the scoping review question was guided by the PCC mnemonic (population, concept, and context) and it will inform inclusion and exclusion criteria and consequently the literature search strategy.

•    Population: health and social care professionals – nursing, medical, allied healthcare professionals/health and social care professionals, health and social care management.

•    Concept: studies whose primary focus is "learning" healthcare system or organisation.

•    Context: studies conducted in rehabilitation healthcare settings.

•    Types of evidence sources: peer-reviewed qualitative, quantitative or mixed-methods empirical studies, reviews, and grey literature in the English language. This includes conference proceedings and opinion pieces. Studies will be included if they define a “learning organisation” or “learning health system” and focus on or specifies relevance to a rehabilitation context. Alternatively, papers will be included if they describe an operating LHS (research focused on LHS data analysed) and/or translation of research evidence generated from LHS data into healthcare improvement, within a rehabilitation context.

Papers will be excluded if they contain no substantive discussion of the learning healthcare system or learning organisation concept or are conducted outside rehabilitation settings. Non-English language studies will be excluded due to time and resources required for translation. Considering the limited number of relevant papers from rehabilitation settings, the authors decided against limiting the search by date. Animal research and poster abstracts will also be excluded.

### Study selection

The found articles will be imported into the bibliographic reference management software Endnote, and any duplicates removed. The systematic review software tool, Covidence, will be used for screening of the retrieved literature. A suitable free alternative to Covidence software is Rayyan. For the pilot testing, a random sample of 25 titles/abstracts will be selected. These will be screened by the entire team using the eligibility criteria and definitions/elaboration document. The reviewers will meet to discuss discrepancies and make modifications to the eligibility criteria and definitions/elaboration document. Team will only start screening when 75% (or greater) agreement is achieved. The reviewers will meet at the beginning, midpoint, and final stages of the abstract review process to discuss challenges and uncertainties related to study selection and to the search strategy if needed. Any disagreements will be resolved by involvement of a third reviewer. The full text article review will be undertaken by the same reviewers using the same method, with the two reviewers reviewing the full texts independently and the third reviewer resolving any conflicts and deciding on final inclusion. The process of study selection, as well as the number of identified, screened, assessed, and included articles will be reported using a PRISMA-ScR flow diagram ( Tricco *et al.*, 2018).

### Data extraction

A draft charting table – for extracting the data addressing the research question – based on JBI Manual for Evidence Synthesis ( Peters *et al.*, 2020) was drafted at the protocol stage (see Table 3). Descriptive data about the study documents will be extracted. This will include:

**Table 3.** Data charting table - draft.

Study ID	Author (s)	Publication date	Journal info	Journal discipline	Country of origin	Research design and methodology	Professionals involved	Research setting/ organisation	Concept/ definition of LHS	Patient inclusion	Implemented changes	Impact of changes

- Author(s)
- Year of publication
- Journal information
- Origin/country of origin (where the source was published or conducted)
- Research design and methodology
- Professionals involved
- What was the setting/organisation for the study?

Data will be extracted under the following headings in order to address the research questions:

- How was the concept of a learning organisation or system defined?
- Who were the participants? Were patients included?
- How were they a learning organisation – what changes were implemented?
- What was the impact of changes made?

The charting table will be continually updated in an iterative process to capture other relevant data that the authors encounter during the process. In line with recommendation from Arksey & O’Malley and JBI Manual for Evidence Synthesis ( Arksey & O'Malley, 2005; Peters *et al.*, 2020), the review team will pilot the data extraction table on a sample of the included studies (10% of the complete list of retrieved studies) to ensure all relevant results are extracted.

### Collating, summarising and reporting the results

A dual approach with frequency counts and qualitative content analysis will be employed to address research questions. Frequency counts will be appropriate for example in demonstrating how many studies focused on operationalising a LO or LHS framework in an organisation, or in ascertaining which journal discipline was most prevalent in the collated studies. Qualitative content analysis will be appropriate to summarise how different papers have conceptualised and defined learning health systems and organisations. Basic coding can be done on definitions using NVivo to identify different ways that the concept has been defined. Coding definitions may result in descriptive themes emerging from the data. Differences and similarities between the concepts of LO and LHS can then be noted by comparing codes/themes arising from each term.

 The results of a scoping review will be presented as a map of the data extracted from the included papers (as charts) and in an aggregate tabular form (as tables) for better visualisation of the results, and in a descriptive format that aligns with the objectives and scope of the review. In line with JBI manual, the qualitative content analysis will be descriptive in nature, as more in-depth analyses such as thematic analysis or synthesis better suited to qualitative evidence syntheses ( Peters *et al.*, 2020). This approach will provide information on the body of research on the concept of “learning” organisations and systems in the context of rehabilitation care, as well as allowing for identification of gaps in the literature.

## Public and patient involvement

This review is part of a larger co-research project. This review team includes healthcare professionals working in rehabilitation as co-researchers and a former rehabilitation patient. Their involvement goes beyond stakeholder consultation, as the co-researchers are contributing not only clinical expertise and knowledge alone but are actively involved in each stage of the review process. All co-researchers have been involved in reviewing the development of this protocol and the conceptual elements of the review question and search.

The involvement of patients and healthcare professionals as knowledge stakeholders has been ensured within this first step of the project. There is evidence that involving patients not only brings lived-experience perspective into research but increases its quality and the relevance ( Brett *et al.*, 2014; Nierse *et al.*, 2012).

The aim is to ensure a democratic co-operation between researchers and involved patients and healthcare professionals. This will include involving them in the design of research and encouraging their uptake of research findings.

## Dissemination

This review will guide a larger research project on teamwork in rehabilitation. The results of the scoping review will be published in a peer reviewed journal and presented at both national and international conferences (see Table 4 for review timeline). All data will be stored in line with best General Data Protection Regulation Practice.

## Timeline for review

**Table 4.** Planned timeline.

Task	Month Start	Estimated Time (months)
Protocol Draft	June 2022	1
Protocol + Search String refine	July 2022	1
Protocol Submit	Aug 2022	1
Review Search	Sept 2022	1
Abstract Screening	Sept 2022	1.5
Full Text Screening	Oct 2022	2
Data Extraction	Nov 2022	1
Analysis	Dec 2022	1
Review Write Up	Jan 2023	2

## Data Availability

No underlying data are associated with this article.
