# Learning healthcare systems and rehabilitation: a scoping review protocol

**DOI:** 10.12688/hrbopenres.13614.3

**Published:** 2026-08-05

**Authors:** Lauren Christophers, Zsofia Torok, Catherine Cornall, Aoife Henn, Clare Hudson, Teresa Whyte, Diarmuid Stokes, Aine Carroll

**Affiliations:** 1 School of Medicine, University College Dublin, Dublin, Ireland; 2 National Rehabilitation Hospital, Dublin, Ireland; 3 UCD Library, University College Dublin, Dublin, Ireland

**Keywords:** Learning healthcare system, learning organisation, rehabilitation, systems thinking, complexity

## Abstract

**Background:**

Healthcare literature has proposed that “learning organisations” (LO) or “learning healthcare systems” (LHS) that continuously generate and apply evidence, innovation, quality, and value can provide better care. This is of value to non-acute healthcare settings such as rehabilitation, which are complex, multi-dimensional and multi-disciplinary in nature. Little is known about how these frameworks have been applied to rehabilitation settings.

**Objective and inclusion criteria:**

The aim of this scoping review is to systematically summarise the literature conceptualising and operationalising LHS and LO in rehabilitation settings. Studies will be included which define a LO or LHS, describe an operating LHS/LO or include the translation of research evidence generated from LHS/LO data into healthcare improvement within a rehabilitation context. All study designs will be included.

**Methods:**

The guidelines from the Joanna Briggs institute methodology for scoping reviews will be used for this review. The literature search will be performed using a three-step search strategy: an initial limited search of two databases has been performed to identify relevant key words and index terms. The developed search string will be adapted and applied across the following databases: OVID MEDLINE, EMBASE, CINAHL Plus, APA PsycINFO and COCHRANE Database of Systematic Reviews. Reference lists of selected sources and relevant data-hubs will also be searched. A draft data extraction framework will be used and updated iteratively to extract data. Data will be extracted and described to address the research question of how LHS and LO have been conceptualised and operationalised in the context of rehabilitation.

**Discussion and Implications:**

There is potential for rehabilitation focused LHSs to advance global rehabilitation services and facilitate best practice. Little is known about how rehabilitation focused LHSs have been constructed and sustained. This will be the first review to explore LHS application in rehabilitation, guiding future development and research.

## Introduction

The increasing aging population, evolving health needs, and rising public expectations for improved health outcomes have placed significant pressure on health systems. Recognizing the crucial role of rehabilitation in strengthening health systems, the World Health Organization (WHO) has introduced the Rehabilitation 2030 initiative highlighting the need for scaling up and enhancing rehabilitation services worldwide ( Krug & Cieza, 2017). With a growing number of individuals living with disabilities ( Cieza, 2019; Krug & Cieza, 2017; WHO, 2020), advancing rehabilitation services has the potential to reduce disability prevalence, decrease healthcare costs ( E. P. a. R. M. B Alliance, 2018), and enable individuals to live longer and better lives.

One model that shows promise in advancing rehabilitation services is the concept of a learning healthcare system (LHS). LHS involves a data-driven approach, where routinely collected healthcare data are utilized to drive continuous improvement and knowledge creation in healthcare practices ( Enticott *et al., *2021). Key features of LHS include cyclical data-driven processes, embedded researchers, academic partnerships, and a focus on being service-led and community-led ( Enticott *et al.*, 2021; Johnson *et al.*, 2021). The roots of the LHS concept trace back to the notion of a “learning organization” (LO) developed in the 1990s by Peter Senge and others ( Senge, 2006). This concept aimed to build adaptive organizations that embrace learning and lead change. While initially applied to private companies, the concept has since been extended to public organizations, including healthcare systems ( Rashman *et al.*, 2009). Extending LO principles in this way requires frameworks that are sufficiently complex and dynamic to accommodate the distinctive characteristics of healthcare delivery.

Acknowledging the complexity of interacting components within and across organisations is known as “systems thinking” and is a key feature of LOs ( Iles & Sutherland, 2001). This is not limited to a single organisation: in rehabilitation in particular, care is frequently delivered across multiple services and providers, so a systems lens must extend beyond any one organisation’s boundaries. In the context of healthcare, applying a complexity lens means recognizing the intricate and multifaceted care networks across different settings, presenting challenges for healthcare delivery ( Braithwaite *et al.*, 2021; Carroll, 2021). LOs also distinguish between different types of learning, such as single loop, double loop, and deutero-learning, each with its own characteristics and objectives ( Argyris & Schön, 1978). Similarly, the concept of a LHS encompasses harnessing routinely collected health data, not necessarily “big data”, for continuous learning and improvement ( Enticott et al., 2021). The core idea for both LHSs and LOs is for health systems and organisations to learn and adapt.

Both LHS and LO concepts share common challenges and criticisms, such as inconsistent terminology ( Enticott *et al.*, 2021; Shea & Taylor, 2017) and a lack of empirical evidence on effective implementation and support ( Budrionis & Bellika, 2016; Huber, 1991; Tosey *et al.*, 2012; Visser, 2007). Despite these challenges, the benefits of LHS models across different healthcare settings are well supported by Enticott *et al.*’s (2021) systematic review. Applying LHS principles to rehabilitation care, with its interdisciplinary nature and focus on patient co-production and goal-setting, could potentially facilitate best practices and provide regular feedback on progress and goals. However, there is a need for further research to explore how LHS models have been applied in rehabilitation contexts and identify key learnings from these applications. Evidence syntheses on LHS have been conducted ( Budrionis & Bellika, 2016; Enticott *et al.*, 2021; McLachlan *et al.*, 2018; Platt *et al.*, 2020; Pomare *et al.*, 2022) but none (as far the authors are aware) have focused on rehabilitation settings.

In summary, the LHS concept promotes data-driven healthcare improvement and draws from the concept of a learning organization. While LHS has been applied in various healthcare settings ( Platt *et al.*, 2020) its implementation in rehabilitation remains limited. This review aims to contribute to the field of healthcare improvement by exploring LHS adoption in rehabilitation and extracting key insights from existing applications. A scoping review is an appropriate type of evidence synthesis for this topic as it will allow the review team to summarise the breadth of evidence available on the topic of LHS/LO in rehabilitation, identify key features of LHS/LO in this context and describe how existing rehabilitation services have become LHS/LO.

### Review question

How have “learning health systems” and “learning organisations” been conceptualised and operationalised in the field of rehabilitation?

The objectives of this review are:

• To gather and note the prevalence of literature which applies LHS and LO concepts to the rehabilitation context.
• From this literature, to collate different definitions of learning healthcare organisations and systems, and note core features of the concepts
• To record how LO or LHS frameworks were applied in rehabilitation settings and the changes made within the organisation.

## Methods

### Screening protocol

The review will be guided by the methodological framework for scoping reviews outlined by Arksey and O’Malley (2005) and Levac *et al.* (2010), and the evidence synthesis guidelines from the Joanna Briggs Institute (JBI) Manual for Evidence Synthesis ( Peters *et al.*, 2020).

### The review team

All co-authors actively participated in the development of this protocol. The full review team consists of three academic researchers, a nurse, a speech and language therapist, a physiotherapist, a rehabilitation hospital programme manager and a university librarian. Three members of the review team (CH, ZT and LC) will undertake the screening of the retrieved literature and the data extraction. Two reviewers will dual screen all abstracts and full texts (CH and ZT), and a third reviewer (LC) will resolve any conflict and decide on final inclusion. The entire process will be supervised by a senior academic researcher who is extensively experienced in scoping review methods (AC).

### Electronic databases

The research team will undertake a comprehensive search of the literature within the following databases:

OVID MEDLINE (1946–9/2022).

EMBASE (1947–9/2022).

CINAHL Nursing and Allied Health (CINAHL Plus) (1961–9/2022).

APA PsycINFO (1967–9/2022).

COCHRANE Database of Systematic Reviews (1996–9/2022).

Each database’s stated inception date is retained as the lower search limit for transparency and completeness. However, as the concepts of learning organisations and learning health systems only emerged from the 1990s onward, few if any relevant records are anticipated from earlier years. To ensure the review captures recent literature, the search will be re-run across all databases immediately prior to full-text screening, and any additional eligible records identified will be incorporated. In addition to these bibliographic databases, grey literature will be identified through hand-searching of two rehabilitation-specific data-hubs (described under Key search terms and search strings, below), backward citation (reference list) searching of included sources, and targeted searching of the web, including Google and Google Scholar, for relevant grey literature such as reports, policy documents, and conference proceedings not indexed in the databases above.

### Key search terms and search strings

The key search concepts for this study are ‘learning health system’ AND ‘rehabilitation’. Alternative and related terms for each of the concepts will also be included, and exclusion terms will be added to eliminate results related to substance abuse and acute care rehabilitation. Table 1 contains the keywords and exclusion terms for the search strings. The search query will be adapted for each database using specific Boolean operators, truncation markers, and MeSH-, Emtree-, subject- and index terms and headings where necessary. The research team collaborated with an expert university librarian (D.S.) in designing and refining the search strategy. Table 2 contains the search strings for each database.

**Table 1.** Keywords for the search strings.

Learning health system	Rehabilitation	Exclude
learning health*	rehabilitati*	addict*
LHS	physiotherapy	alcohol *
rapid-learning	physical therapy	substance
RLHS	occupational therapy	abuse
learning organisation	neurorehabilitation	misuse
systems thinking	habilitation	mental health
		cardiac
		pulmonary
		narcotic*
		psychiatry*

**Table 2.** Search strings for each database.

Database	Core search (title, abstract)	MeSH-, Emtree-, subject- or index terms
OVID MEDLINE	Learning health* or LHS or Rapid-learning or RLHS or Learning organisation or systems thinking and Rehabilitati* or physiotherapy or physical therapy or occupational therapy or neurorehabilitation or habilitation	exp Learning Health System/ or *Organizational Innovation/and *Rehabilitation/ or *Hospitals, Rehabilitation/ or *"Physical and Rehabilitation Medicine”/ or *Stroke Rehabilitation/ or *Neurological Rehabilitation/ or *Rehabilitation Centers/or *Rehabilitation Research/
EMBASE	‘learning health*’ OR lhs OR ‘rapid learning’ OR rlhs OR ‘learning organisation’ OR ‘systems thinking’ AND rehabilitati* OR physiotherapy OR ‘physical therapy’ OR ‘occupational therapy’ OR neurorehabilitation OR habilitation	‘learning health system’/exp AND ‘stroke rehabilitation’/mj OR ‘occupational therapy’/mj OR ‘sensorimotor integration’/mj OR ‘speech and language rehabilitation’/mj OR ‘neurorehabilitation’/mj
CINAHL	“Learning health*” OR LHS OR Rapid-learning OR RLHS Or “Learning organisation” OR “Learning Based” OR “systems thinking” AND Rehabilitati* OR physiotherapy OR “physical therapy” OR “occupational therapy” OR neurorehabilitation OR habilitation	MM “Learning Health System” AND MM “Activities of Daily Living+”) OR (MM “Rehabilitation”) OR (MM “Occupational Therapy”) OR (MM “Physical Therapy”
PsycINFO	“Learning health*” OR Rapid-learning OR RLHS OR “Learning organisation” OR “systems thinking” AND Rehabilitati* OR physiotherapy OR “physical therapy” OR “occupational therapy” OR neurorehabilitation OR habilitation	subject(“Organizational Learning “) AND MJMAINSUBJECT.EXACT(“Rehabilitation”) OR MJMAINSUBJECT. EXACT(“Rehabilitation Centers”) OR MJMAINSUBJECT.EXACT (“Neurorehabilitation”) OR MJMAINSUBJECT.EXACT(“Vocational Rehabilitation”) OR MJMAINSUBJECT.EXACT(“Telerehabilitation”)
COCHRANE	“Learning health*” OR LHS OR Rapid-learning OR RLHS Or “Learning organisation” OR “Learning Based” OR “systems thinking” AND Rehabilitati* OR physiotherapy OR “physical therapy” OR “occupational therapy” OR neurorehabilitation OR habilitation	[Learning Health System] OR [Organizational Innovation] AND [Rehabilitation] OR [Rehabilitation Centers] OR [Hospitals, Rehabilitation] OR [Physical and Rehabilitation Medicine] OR [Rehabilitation of Speech and Language Disorders] OR [Rehabilitation Research] OR [Neurological Rehabilitation] OR [Telerehabilitation] OR [Stroke Rehabilitation]

As recommended by the JBI Manual for Evidence Synthesis ( Peters *et al.*, 2020), a three-step process for applying a search strategy will be implemented. Step 1 has already been carried out and involved an initial limited search on multiple databases for relevant articles. This has been followed by analysis of the keywords and phrases contained in the titles and abstracts of the retrieved papers, and of the index terms used to describe the articles. Step 2 will involve the search across all included databases using the identified key words outlined in Table 1. Step 3 will involve the search of the reference lists of selected sources to identify any additional relevant studies, as well as hand search of two rehabilitation datahubs involved in application of LHSs, the International Spinal Cord Injury Community Survey (InSCI) ( http://www.swisci.ch) and the LeaRRn|Learning Health Systems Rehabilitation Research Network (LeaRRn) ( sites.brown.edu/learrn/), and targeted grey literature searching as described above.

### Inclusion/exclusion criteria

In line with methodological framework by Arksey and O’Malley (2005), the final inclusion and exclusion criteria will be devised post hoc, based on increasing familiarity with the literature. Following the JBI guidelines, however, the scoping review question was guided by the PCC mnemonic (population, concept, and context) and it will inform inclusion and exclusion criteria and consequently the literature search strategy.

• Population: health and social care professionals – nursing, medical, allied healthcare professionals/health and social care professionals, health and social care management.
• Concept: studies whose primary focus is “learning” healthcare system or organisation.
• Context: studies conducted in rehabilitation healthcare settings.
• Types of evidence sources: peer-reviewed qualitative, quantitative or mixed-methods empirical studies, reviews, and grey literature in the English language. This includes conference proceedings and opinion pieces. Studies will be included if they define a “learning organisation” or “learning health system” and focus on or specifies relevance to a rehabilitation context. Alternatively, papers will be included if they describe an operating LHS (research focused on LHS data analysed) and/or translation of research evidence generated from LHS data into healthcare improvement, within a rehabilitation context.

Papers will be excluded if they contain no substantive discussion of the learning healthcare system or learning organisation concept or are conducted outside rehabilitation settings. Non-English language studies will be excluded due to time and resources required for translation. Considering the limited number of relevant papers from rehabilitation settings, the authors decided against limiting the search by date. Animal research and poster abstracts will also be excluded. In line with the guidelines from the Joanna Briggs Institute (JBI) Manual for Evidence Synthesis and Levac ( Levac *et al.*, 2010; Peters *et al.*, 2020), at the beginning of the process, the team will meet to discuss decisions surrounding study inclusion and exclusion. Two reviewers will dual screen all abstracts (see Study selection, below, for full details of conflict resolution).

### Study selection

The found articles will be imported into the bibliographic reference management software Endnote ( https://endnote.com/), and any duplicates removed. The systematic review software tool, Covidence ( www.covidence.org), will be used for screening of the retrieved literature. A suitable free alternative to Covidence software is Rayyan ( https://www.rayyan.ai/).For the pilot testing, a random sample of 25 titles/abstracts will be selected. These will be screened by the entire team using the eligibility criteria and definitions/elaboration document. The reviewers will meet to discuss discrepancies and make modifications to the eligibility criteria and definitions/elaboration document. Team will only start screening when 75% (or greater) agreement is achieved. The reviewers will meet at the beginning, midpoint, and final stages of the abstract review process to discuss challenges and uncertainties related to study selection and to the search strategy if needed. Any disagreements will be resolved by involvement of a third reviewer. The full text article review will be undertaken by the same reviewers using the same method, with the two reviewers reviewing the full texts independently and the third reviewer resolving any conflicts and deciding on final inclusion. The process of study selection, as well as the number of identified, screened, assessed, and included articles will be reported using a PRISMA-ScR flow diagram ( Tricco *et al.*, 2018).

### Data extraction

A draft charting table – for extracting the data addressing the research question – based on JBI Manual for Evidence Synthesis ( Peters *et al.*, 2020) was drafted at the protocol stage (see Table 3). Descriptive data about the study documents will be extracted. This will include:

• Author(s)
• Year of publication
• Journal information
• Origin/country of origin (where the source was published or conducted)
• Research design and methodology
• Professionals involved
• What was the setting/organisation for the study?

**Table 3.** Data charting table - draft.

Study ID	Author(s)	Publication date	Journal info	Journal discipline	Country of origin	Research design and methodology	Professionals involved	Research setting/ organisation	Concept/ definition of LHS	Patient inclusion	Implemented changes	Impact of changes
												
												
												
												
												
												

Data will be extracted under the following headings in order to address the research questions:

• How was the concept of a learning organisation or system defined?
• Who were the participants? Were patients included?
• How were they a learning organisation – what changes were implemented?
• What was the impact of changes made?

The charting table will be continually updated in an iterative process to capture other relevant data that the authors encounter during the process. In line with recommendation from Arksey & O’Malley and JBI Manual for Evidence Synthesis ( Arksey & O’Malley, 2005; Peters *et al.*, 2020), the review team will pilot the data extraction table on a sample of the included studies (10% of the complete list of retrieved studies) to ensure all relevant results are extracted.

### Collating, summarising and reporting the results

A descriptive approach will be employed to address research questions. Frequency counts will be appropriate for example in demonstrating how many studies focused on operationalising a LO or LHS framework in an organisation, or in ascertaining which journal discipline was most prevalent in the collated studies. Basic coding can be done on definitions using NVivo ( https://www.qsrinternational.com/nvivo-qualitative-data-analysis-software/) to identify different ways that the concepts have been defined, and key features of the concepts can be listed. The results pertaining to how the LHS or LO concept was *applied* to the rehabilitation setting will be descriptively summarised on a paper by paper basis.

The results of a scoping review will be presented as a map of the data extracted from the included papers (as charts) and in an aggregate tabular form (as tables) for better visualisation of the results, and in a descriptive format that aligns with the objectives and scope of the review. In line with JBI guidance for scoping reviews, the data analysis undertaken in this review will be descriptive rather than interpretive in nature (for example, presenting frequency counts and a narrative summary of extracted data), as more in-depth analytic approaches such as thematic analysis or synthesis are better suited to qualitative evidence syntheses ( Peters *et al.*, 2020). This approach will provide information on the body of research on the concept of “learning” organisations and systems in the context of rehabilitation care.

### Team composition

This review is part of a larger co-research project. The review team is multidisciplinary and includes academic researchers alongside healthcare professionals working in rehabilitation; nursing, speech and language therapy, physiotherapy, and rehabilitation programme management. Their involvement goes beyond stakeholder consultation: these co-researchers contribute clinical expertise and are actively involved in each stage of the review process, and all are credited as co-authors. We note that we do not characterise the involvement of these healthcare professional co-researchers as patient and public involvement (PPI); rather, it reflects the multidisciplinary composition of the review team itself.

## Public and patient involvement

This scoping review protocol is part of a larger co-research project, within which a person with lived experience of rehabilitation; a former patient of the National Rehabilitation Hospital, has been an integral co-researcher since the project’s inception. This patient partner’s reflections and priorities, shared at regular co-research team meetings, directly informed the decision to prioritise this scoping review as a first step in the wider programme of work on teamwork in rehabilitation; in this sense, their input shaped the existence and direction of this protocol, even though their contribution to this specific review has, to date, been made through the wider project rather than through direct authorship of this article. They are not a co-author of the present protocol but will continue to be consulted as the review progresses, and may be invited to co-author future outputs from the wider co-research project.

## Dissemination

This review will guide a larger research project on teamwork in rehabilitation. The results of the scoping review will be published in a peer reviewed journal and presented at both national and international conferences (see Table 4 for review timeline). All data will be stored in line with best General Data Protection Regulation Practice.

## Discussion & Implications

The creation and implementation of rehabilitation-focused LHSs offers great potential for rehabilitation services, which are currently in global need of advancement and development. Rehabilitation is a holistic process involving interdisciplinary collaboration and active involvement of patients throughout their care journey. Rehabilitation services thus exemplify co-production with service users ( Batalden *et al.*, 2016). Another key aspect of rehabilitation care is goal setting ( NICE, 2009; Stroke Foundation, 2017), but contrary to best practice, there is often a lack of review, feedback, and goal breakdown ( Plant & Tyson, 2018; Scobbie *et al.*, 2015). LHS principles align with the interdisciplinary nature of rehabilitation, co-production, and the need for progress monitoring and continuous feedback. By applying LHS in rehabilitation settings, best practices could be facilitated to improve patient outcomes and experience.

This review is not without anticipated methodological challenges. As previously reported by other evidence syntheses in this field ( Budrionis & Bellika, 2016; Enticott *et al.*, 2021), inconsistent terminology and the absence of an agreed definition of LHS and LO make comprehensive literature searching and screening difficult; our broad, non-date-limited search strategy, developed with an expert librarian and refined through iterative piloting of the eligibility criteria, is intended to mitigate this risk. We also anticipate that rehabilitation-focused literature on this topic will be sparse and heterogeneous in design and reporting quality, which is why we have adopted a descriptive rather than interpretive analytic approach consistent with JBI scoping review guidance.

This review will extend beyond Enticott *et al.*’s (2021) systematic review, which examined the benefits of LHS across healthcare settings broadly, by providing the first context-specific account of how LHS and LO concepts have been defined and operationalised specifically within rehabilitation, a setting distinguished by its interdisciplinary team-based delivery, emphasis on patient co-production, and goal-oriented model of care, none of which were a specific focus of that prior review.

This review will examine the application of LHS and LO concepts in rehabilitation settings, contributing to the understanding of how rehabilitation focused LHS are developed and operationalized. Findings from this review can be used to inform the development of future rehabilitation LHSs and direct the way for further research on the concept.

**Author roles**
**Christophers, L:** Conceptualization, Investigation, Methodology, Project Administration, Resources, Supervision, Visualization, Writing – Original Draft Preparation, Writing – Review & Editing; **Torok, Z:** Conceptualization, Investigation, Project Administration, Visualization, Writing – Original Draft Preparation, Writing – Review & Editing; **Cornall, C**: Conceptualization, Resources, Writing – Review & Editing; **Henn, A**: Conceptualization, Resources, Writing – Review & Editing; **Hudson, C:** Conceptualization, Resources, Writing – Review & Editing; **Whyte, T:** Conceptualization, Resources, Writing – Review & Editing; **Stokes, D:** Investigation, Methodology, Resources; **Carroll, Á:** Conceptualization, Funding Acquisition, Methodology, Resources, Supervision, Writing – Review & Editing.

## Data availability

### Underlying data

No underlying data are associated with this article.
