# Supplementary material for: Learning healthcare systems and rehabilitation: a scoping review protocol
Source: HRB Open Res. 2026 Aug 5;5:65. Originally published 2022 Oct 11. [Version 3] doi: 10.12688/hrbopenres.13614.3 (PMC13562931; doi:10.12688/hrbopenres.13614.3)
Supplement: Peer review history

# Reviewer report: Reviewer response for version 3

**DOI:** 10.21956/hrbopenres.15994.r57138

**Published:** 2026-08-31

**Authors:** Vanessa Abrahamson

**Affiliations:** 1 University of Kent, Canterbury, England, UK

**Overall: **this is a worthwhile review with clear rationale and relevance to those who work in rehabilitation. The other reviewers have provided comprehensive feedback so I will not reiterate their helpful comments which have already been addressed.

 It may be worthwhile clarifying earlier on that rehab covers inpatient settings. It is clear that acute settings are excluded but I was initially unsure if community rehab (in the person’s home) was included.

 I notice that the team does not include an occupational therapist which would be a useful addition.

**Abstract: **clear aim and valid rationale, covers the key elements of the method. No further comments.

**Introduction**: clear structure, covers useful background information, what the concept of learning healthcare system (LHS) means and common criticisms. Their rationale is clearly stated, the aim is clear and choice of a scoping review appears appropriate.

 Re the **second objective**, ‘to collate different definitions of learning healthcare organisations and systems, and note core features of the concepts’ – noting core features is likely to be difficult to capture.

**Data extraction**: following on from the above comment, Table 3, I can see that the column ‘concept/definition of LHS’ will incorporate the definition and the core features, but it might be worth while to separate into two columns so that the *features* don’t get overlooked. Obviously, you can refine your table according to what works once you start data extraction.

**Search terms** in Table 1 are comprehensive and I’m sure carefully piloted. I noticed truncation of rehabilitation but not of physiotherapy or occupational therapy (e.g. therap* in include therapy and therapist). The search strings in Table 2 are comprehensive.

**Collating, summarising and reporting the results**: this has been thought through and seems appropriate.

 Thank you for sharing this interesting and comprehensive protocol, I look forward to reading the findings in due course.

Is the study design appropriate for the research question?

Yes

Is the rationale for, and objectives of, the study clearly described?

Yes

Are sufficient details of the methods provided to allow replication by others?

Yes

Are the datasets clearly presented in a useable and accessible format?

Not applicable

Reviewer Expertise:

primary and community care; rehabilitation; older people; stroke; end-of-life care; building research capacity in health and social care

I confirm that I have read this submission and believe that I have an appropriate level of expertise to confirm that it is of an acceptable scientific standard.

# Reviewer report: Reviewer response for version 2

**DOI:** 10.21956/hrbopenres.15071.r35758

**Published:** 2023-10-02

**Authors:** Christian von Plessen

**Affiliations:** 1 Center for Primary Care and Public Health (Unisanté), University of Lausanne, Lausanne, Switzerland

Thank you for inviting me to comment on the revised version of this very interesting protocol. I am looking forward to reading the results of this study. 

 I only have a few minor comments to add. 

1. Introduction, first para.: "...strengthening healthcare systems,..." Is it not strengthening "health" rather than strengthening "health care systems"?
2. "This requires frameworks..." Are you referring to the need for a framework that can accommodate (or be useful given) the complexity of health care? 
3. Second para.: "...within an organization...". I would not limit systems thinking to "within an organization". It is indeed also useful when more than one organization is involved, which often is the case in rehabilitation. 
4. "...the power of big data..." I find this term that is often used in marketing not very clear. Also LHS initiatives use any data (aggregated) not only big data. 
5. "Electronic databases" section. There will be hardly any literature on your topic in the years before the first publications on learning organizations. So, starting in 1946 will probably yield little, but you miss out on interesting articles when you stop in September 2022. Also, I wonder how you will search for grey literature in the mentioned databases. What about websites, Google etc.?
6. Study selection section. Repetition from last para. of section before: "Any disagreement will be..."
7. Collating, summarizing and reporting the results section, second para. What do you mean by "manual analysis will be descriptive in nature"?
8. Public and patient involvement section. The involvement of health care professionals is usually not considered PPI. Delete the first paragraph? The second paragraph describes the role of a patient. You emphasize the importance of his/her input ("important", "integral", "invaluable", "greatly"), but the nature of the implication and the background of the person remains unclear. Please consider clarifying this. 
9. Discussion and implications section. 
  You argue well for why LHS make sense in rehabilitation, but what about other aspects of this study? Methodological challenges? What do you expect to add beyond the review by Enticott et al.?

I hope these comments are helpful and I wish you good luck with this important work.

Is the study design appropriate for the research question?

Yes

Is the rationale for, and objectives of, the study clearly described?

Partly

Are sufficient details of the methods provided to allow replication by others?

Yes

Are the datasets clearly presented in a useable and accessible format?

Not applicable

Reviewer Expertise:

Quality improvement, patient safety, coproduction, organisational learning.

I confirm that I have read this submission and believe that I have an appropriate level of expertise to confirm that it is of an acceptable scientific standard, however I have significant reservations, as outlined above.

# Response

**Published:** 2026-07-06

**Authors:** Áine Carroll

**Affiliations:** University College Dublin, Ireland

**Response to Christian von Plessen (Reviewer Report, 02 October 2023)**

***Reviewer comment:*** *Introduction, first paragraph: “...strengthening healthcare systems,...” Is it not strengthening “health” rather than strengthening “health care systems”?*

**Author response: **We agree and have corrected this to align with the WHO's own terminology in the Rehabilitation 2030 initiative. The sentence now reads “recognizing the crucial role of rehabilitation in strengthening health systems”.

***Reviewer comment:*** *“This requires frameworks...” Are you referring to the need for a framework that can accommodate (or be useful given) the complexity of health care?*

**Author response: **Yes; we have reworded this sentence for clarity so that the referent is explicit: extending learning organisation principles to public-sector and healthcare organisations requires frameworks that are sufficiently complex and dynamic to accommodate the distinctive characteristics of healthcare delivery.

***Reviewer comment:*** *Second paragraph: “...within an organization...” I would not limit systems thinking to “within an organization”. It is indeed also useful when more than one organization is involved, which often is the case in rehabilitation.*

**Author response: **This is a helpful point, particularly given the interdisciplinary and often multi-agency nature of rehabilitation services. We have revised this sentence to state that systems thinking involves acknowledging the complexity of interacting components within and across organisations, and have added a brief note that rehabilitation care frequently spans multiple providers and services.

***Reviewer comment:*** *“...the power of big data...” I find this term that is often used in marketing not very clear. Also LHS initiatives use any data (aggregated) not only big data.*

**Author response: **We agree the term was imprecise and have removed it. The sentence now refers to LHS harnessing routinely collected health data, rather than “big data” specifically, for continuous learning and improvement, with a citation to Enticott et al. (2021).

***Reviewer comment:*** *“Electronic databases” section. There will be hardly any literature on your topic in the years before the first publications on learning organizations. So, starting in 1946 will probably yield little, but you miss out on interesting articles when you stop in September 2022. Also, I wonder how you will search for grey literature in the mentioned databases. What about websites, Google etc.?*

**Author response: **We have clarified that database inception dates are retained as the lower search limit for completeness and transparency, rather than an expectation of relevant early yield, since the concepts of learning organisations and learning health systems only emerged from the 1990s onward. We have also added a statement that the database search will be re-run prior to full-text screening to capture literature published since the original search date, and clarified that grey literature will be captured through hand-searching of the two named rehabilitation data-hubs, reference list (snowball) searching, and targeted searching of the web (including Google/Google Scholar) for relevant grey literature such as reports and policy documents, in addition to the named bibliographic databases.

***Reviewer comment:*** *Study selection section. Repetition from last paragraph of section before: “Any disagreement will be...”*

**Author response: **We have removed the duplicated sentence regarding conflict resolution from the Inclusion/exclusion criteria section, retaining the fuller description of this process in the Study selection section only.

***Reviewer comment:*** *Collating, summarizing and reporting the results section, second paragraph. What do you mean by “manual analysis will be descriptive in nature”?*

**Author response: **We have reworded this sentence, which was unclear as written. It now reads that, in line with JBI guidance for scoping reviews, the data analysis undertaken will be descriptive rather than interpretive in nature (for example, presenting frequency counts and a narrative summary of extracted data), as more in-depth analytic approaches such as thematic analysis or synthesis are better suited to qualitative evidence syntheses (Peters et al., 2020).

***Reviewer comment:*** *Public and patient involvement section. The involvement of health care professionals is usually not considered PPI. Delete the first paragraph? The second paragraph describes the role of a patient. You emphasize the importance of his/her input (“important”, “integral”, “invaluable”, “greatly”), but the nature of the implication and the background of the person remains unclear. Please consider clarifying this.*

**Author response: **We agree with this distinction and have restructured the section into two parts: “Team composition”, describing the multidisciplinary co-researcher team of healthcare professionals (who are credited as co-authors and whose involvement we no longer describe as PPI), and “Patient and public involvement”, which is now specific to our patient partner. We have clarified that this patient partner is a former patient of the National Rehabilitation Hospital with lived experience of rehabilitation, and specified concretely how their input shaped the wider co-research project, namely that their reflections at co-research meetings directly informed the decision to prioritise this scoping review as a first step in the programme of work. We have also clarified why they are not currently a co-author on this specific output, while noting their continued involvement in the wider project.

***Reviewer comment:*** *Discussion and implications section. You argue well for why LHS make sense in rehabilitation, but what about other aspects of this study? Methodological challenges? What do you expect to add beyond the review by Enticott et al.?*

**Author response: **We have added a new paragraph to this section addressing anticipated methodological challenges, particularly inconsistent terminology and definitional ambiguity in the LHS/LO literature (as previously reported by Budrionis & Bellika, 2016 and Enticott et al., 2021), and the likely sparsity and heterogeneity of rehabilitation-focused literature, which limits us to descriptive rather than interpretive synthesis. We have also explicitly distinguished the contribution of this review from Enticott et al. (2021): whereas that systematic review examined LHS across healthcare settings broadly, this scoping review will provide a context-specific account of how LHS and LO concepts have been defined and operationalised specifically within rehabilitation, a setting with distinctive features (interdisciplinarity, patient co-production, and goal-based care) not addressed in that prior work.

 We believe these revisions fully address the reservations raised and have strengthened the clarity and rigour of the protocol. We thank the reviewer for their generous and helpful input.

# Reviewer report: Reviewer response for version 2

**DOI:** 10.21956/hrbopenres.15071.r35759

**Published:** 2023-08-17

**Authors:** Marissa Bird

**Affiliations:** 1 Institute for Better Health, Trillium Health Partners, Mississauga, Ontario, Canada

Thank you for the opportunity to re-review this article. I believe that the authors have done an excellent job at incorporating the suggested revisions, enhancing the clarity and conciseness of their article, and emphasizing the implications of this work. I have no further comments to add, other than to congratulate authors on an excellent contribution.

Is the study design appropriate for the research question?

Yes

Is the rationale for, and objectives of, the study clearly described?

Yes

Are sufficient details of the methods provided to allow replication by others?

Partly

Are the datasets clearly presented in a useable and accessible format?

Not applicable

Reviewer Expertise:

My work is in healthcare innovation in learning health systems, with specific foci on co-design and evaluation of healthcare innovations.

I confirm that I have read this submission and believe that I have an appropriate level of expertise to confirm that it is of an acceptable scientific standard.

# Reviewer report: Reviewer response for version 1

**DOI:** 10.21956/hrbopenres.14885.r34309

**Published:** 2023-06-22

**Authors:** Marissa Bird

**Affiliations:** 1 Institute for Better Health, Trillium Health Partners, Mississauga, Ontario, Canada

Thank you for the opportunity to review this work. This is an important topic with implications for moving the field of rehabilitation-focused Learning Health Systems forward. What follows is a summary of this work and suggestions for areas to add clarity and strength to this scoping review protocol.

**Summary:**

 Authors Christophers and colleagues present a protocol for a scoping review focusing on the concept of rehabilitation-focused Learning Healthcare Systems. The authors present an introduction with some detail around the concept of Learning Organizations and the application of complexity and systems thinking to the topic of healthcare, as well as Learning Healthcare Systems before stating their aim of contributing knowledge to how healthcare organizations can become learning organizations. The authors detail the methods of their review next, and conclude with a section on dissemination.

 I have identified a number of major and minor comments which I believe will help to strengthen this review and add clarity in a number of places.

**Major comments:**

*Introduction:*

 The introduction is thorough, however, I would ask the authors to consider stating up front the challenge they are attempting to tackle in this work before proceeding into more detail by elaborating on Learning Organizations, complexity, and systems thinking. This will make it clear for the reader what to expect in terms of content and topic for the work at hand.

 To make this section a bit tighter, I think you could remove the entire first paragraph on integrated care. While this is useful context, it’s not really the primary focus of your review.

 As well, I would tie in the focus on rehabilitation early on in the introduction. Its inclusion near the end of this section at present seems to position it as an afterthought, when in reality it is a central concept to your work.

*Review question and objectives:*

 The review question, and at least two of the four objectives, focus on the application of LO and LHS concepts to the field of rehabilitation. If this is the primary objective of this review (which I believe is a gap and a great place to focus), I would suggest removing Objective 2, of comparing and contrasting LO and LHS definitions. Authors provide a thorough introduction comparing these two concepts as well as their histories, and I believe including the compare/contrast exercise in the review results will detract from the primary focus of the application of these concepts within the field of rehabilitation.

 Secondly, if you wish to include Objective 4, I would add more detail on how impact will be measured and the types of indicators you will consider (strictly quantitative impact? Economic evaluation? Qualitative evidence? Third party evaluation? Etc.). Otherwise, you could subsume this objective within the one above detailing the application of LO/LHS to rehabilitation settings.

*Methods:*

 If Qualitative Content Analysis is chosen as the analytic method, much more detail needs to be provided on how it will be employed and who will be applying it. For example, authors should specify key references guiding their work, the team members involved, and the stages of analysis. However, as this study is a scoping review, I would actually suggest that content analysis is not needed, and authors can simply descriptively summarize similarities and differences across studies.

*Public and patient involvement:*

 It would be helpful to specify the degree of involvement of patient partners. For example, what input was considered from patient partners, and were any substantive changes made to the protocol as a result of their involvement? Are any of the named authors on this review partners? Will patient partner involvement in this work be evaluated in some way?

*Suggested section: Implications*

 I would suggest that authors consider contextualizing their work in the broader field of LHS. This is an important topic and bears implications for how rehabilitation-focused LHS are constructed and operationalized. While the dissemination plans state that this work will guide a larger project, what I think is missing is consideration of how this work will move the field forward in general. Perhaps authors could consider tying this back to the field of rehabilitation and current gaps in learning within that field.

**Minor comments:**

- In the abstract, the second sentence includes the phrase “will be included” twice. The sentence after could be phrased “all study designs will be included”.
- In the introduction, I would suggest removing the sentence listing Senge’s five pillars of learning, as each of these terms, should you choose to include them, probably need their own definition as well. Instead, you could simply choose to emphasize that Senge incorporates ‘systems thinking’ which aligns with complexity theory.
- Consider adding a more fulsome definition of ‘complex adaptive systems’ – this is a nuanced field that not all readers will be familiar with.
- I would consider revising the transition between the following sentences: “Thus, systems thinking is useful in healthcare innovation as it incorporates a complexity lens and complexity sensitive solutions to problems in healthcare. However, common criticisms of “learning organisations” as a concept are that there are contradictory definitions and vague concepts.” I am not sure that there is a clear reason for the use of “however” here.
- The following sentence could be revised for clarity: “With data driven approaches that have gained popularity, particularly in the US, the success is dependent on the existence of electronic health records and infrastructure.”
- In the first methods paragraph, I suggest removing the text on the screening protocol and placing it after inclusion/exclusion criteria.
- Please specify that all databases listed will be searched from inception to [insert month/year].
- I think you could revise your first sentence under “Key terms and search strings” to replace “learning organization” with “learning health system” so as to match Table 1.
- Please clarify the term “relevant data hubs”. Does this refer to publicly accessible data repositories? If so, which ones will be searched?
- I am not sure why the following sentence was included: “A suitable free alternative to Covidence software is Rayyan.” It seems non-applicable as Covidence was specified as the screening software of choice.

Is the study design appropriate for the research question?

Yes

Is the rationale for, and objectives of, the study clearly described?

Yes

Are sufficient details of the methods provided to allow replication by others?

Partly

Are the datasets clearly presented in a useable and accessible format?

Not applicable

Reviewer Expertise:

My work is in healthcare innovation in learning health systems, with specific foci on co-design and evaluation of healthcare innovations.

I confirm that I have read this submission and believe that I have an appropriate level of expertise to confirm that it is of an acceptable scientific standard, however I have significant reservations, as outlined above.

# Reviewer report: Reviewer response for version 1

**DOI:** 10.21956/hrbopenres.14885.r33023

**Published:** 2022-11-08

**Authors:** Christian von Plessen

**Affiliations:** 1 Center for Primary Care and Public Health (Unisanté), University of Lausanne, Lausanne, Switzerland

Thank you for inviting me to review the manuscript *Conceptualising learning healthcare systems and organisations in the context of rehabilitation: a scoping review protocol*.

 The authors present today’s healthcare challenges (aging population, fragmented systems etc.), the need for integrated care and the profound challenge of implementing changes. Then, they describe the origins of the concept of learning organisations (LO) and the need for and the challenges of adaptations to public service. Further, they argue for using systems thinking to understand and to address the complex challenges of health care. They proceed to present the learning health system (LHS) that builds on the concept of Los. There is currently no generally accepted definition of a LHS but some common features can be identified such as data feedback loops, learning focus and community involvement.

 Despite the conceptual appeal of LHS, implementation has proven difficult. A recent systematic review by Enticott *et al.,* (2021) ^1^ documents benefit of LHS across settings. However, no such review exists for rehabilitation medicine. This field, the authors argue, is especially suitable for studying LOs because integrative and person-centred nature of rehabilitation.

 The field of LO has been criticised for imprecise definitions and for lack of empirical evidence. The authors propose to create clarity by focusing on a specific area, rehabilitation and by summarizing the available evidence. Thus, the aims of the systematic review are to (1) summarize the literature on LOs and LHS in rehabilitation, (2) clarify definitions, (3) describe how the concepts were applied and which organisational changes were made, and (4) what the impact of the changes were.

 The authors plan to follow the Joanna Briggs Institute recommendations for systematic reviews. They will search the commonly used literature databases and they present inclusion and exclusion criteria for the studies (Table 1). Detailed search strings for the different databases are presented in Table 2. A pilot review of 25 studies is planned. Studies for the pilot and for the final review will be read by two independent researchers with the implication of a third researcher to resolve disagreements. The authors plan to use a analytical software for literature reviews, for example, Covidence®. They also present an overview over data that will be extracted (Table 3) and they will prepare a PRISMA-ScR flow diagram. They propose qualitative content analysis to extract the meaning of different definitions of LOs and LHS. A former rehabilitation patient will participate in the review process. The review is a part of a larger research project on teamwork in rehabilitation.

 I want to congratulate the authors on their ambitious project which I find interesting and relevant. The terms LOs and in health care especially LHS have emerged as promising ideas. The proposition of contextualizing, clarifying, and summarizing the approaches and results for a specific area such as rehabilitation is a relevant and original idea that should be ripe for a literature review. I also find the choice of systematic review adequate given the current state of knowledge. I have a couple of major and minor comments that I hope will help the authors to improve their study.

**Major comments**

 To further improve the protocol, I would recommend revising the structure of the introduction. In its current form links between integrated care, patient centred care, LOs and LHS are not clear. This leaves the reader with a vague notion about the rationale for the study and for the choice of focus on rehabilitation.

 Second, the authors might consider coming to the point of the study earlier in the introduction, which in its current form is rather long. Stating that LHS have been proposed as a solution to many of today’s challenges might be a way. An explanation of the origins of LHS in LOs follow and then a presentation of common—and generally accepted—elements of LOs and LHS without having to present a univocal definition. Moreover, the authors might want to underline the importance of learning (single and double loop) in LOs. This might help build a bridge to the emphasis on data and feedback in LHS.

 Third, I wonder whether complexity and systems thinking, albeit their conceptual importance, warrant to be presented in as much detail. Given the objectives of the study the focus should be on features of LHS and on the impact of its use.

 Fourth, the authors might consider pointing out more clearly the role of persons sometimes called patients in rehabilitation and LHS. This might make the link between care integration and LHS clearer and it would also be an additional argument for the focus on rehabilitation as an archetypical example of coproduced healthcare service (Batalden *et al*. 2015 ^2^).

 Finally, the rationale of the study would be strengthened by a more detailed presentation of knowledge gaps in the currently available literature, for example a more in depth presentation of the study Enticott *et al*. (2021) ^1^.

**Minor comments**

*Introduction*

 The study by Kruk *et al*. (2018) is on low- and middle-income countries. Is it relevant for the focus of this review?

 The sentence beginning “A potential transformation to healthcare systems […].” sounds a slightly odd. Do you mean that LHS have the potential to contribute to a transformation of health care?

 This study by Rashman *et al.,* (2009) is interesting, but it is not clear from the manuscript why public organisations require more complex and dynamic LO frameworks. In addition, it sounds as if LO and LHS were limited to public health care organisations.

 The sentence “Healthcare systems are characterised by being complex. This complexity emerges from multiple independent agents interconnected in a surrounding environment, with continuous evolution and adaptation – thus healthcare systems qualify as complex adaptive systems.” Resembles a circular definition, i.e., healthcare is complex because it is complex. Please clarify and simplify (if you want to keep this part in the article). Also, you might consider often cited work on complex adaptive systems, for example, Braithwaite J *et al.*, 2021 ^3^.

 The sentence “Thus, systems thinking is useful in healthcare innovation as it incorporates a complexity lens and complexity sensitive solutions to problems in healthcare.” seems to be the conclusion of the preceding paragraph. Why do you use it to open the next?

 Eliminating redundancies and a more precise language would help the reader follow the arguments. For example, in the paragraph beginning “Barriers to the successful implementation of these models largely relate to financial obstacles and infrastructural impediments.” the sentences “However, there has been no review published focused on learning health systems within the specific context of rehabilitation. LHS is a concept which has been used in a wide variety of settings including oncology, surgery, cardiology, primary care and paediatrics (Platt *et al*., 2020), but far more rarely in rehabilitation setting.” could be shortened to, for example: LHS have been studied in a variety of settings but to our knowledge there is no review of the concept in rehabilitation.

 In the three paragraphs preceding the review question, the authors argue for LHS in rehabilitation. Making them shorter and more to the point would help clarify the rationale of the study. For example: The principles of LHS match the complex interdisciplinary nature of rehabilitation, its coproduction by users and professionals, as well as the need to generate and feedback data on progress etc….

*Review question*

 The authors have identified empirical proof of impact of LOs and LHS an important knowledge gap. Please consider adding this term to the review question.

 Also, later in the methods they propose to identify gaps in the literature. Should that be stated as an aim of the review?

*Methods*

 Please consider adding references to tools such as Covidene or NVIVO.

 Please consider adding information about the reviewers. What are their backgrounds, review experiences etc.

 Please add a reference to qualitative content analysis.

 Page 7. The sentence “In line with JBI manual, the qualitative content analysis will be descriptive in nature, as more in-depth analyses such as thematic analysis or synthesis better suited to qualitative evidence syntheses.” seems to lack a verb in the second part. Please revise.

 I have tried the search string for Ovid Medline, but it did not work. Please revise.

*Patient and public involvement*

 The authors mention a former rehabilitation patient in the research team. What is their role? Which contributions have already been made? Specifying this might be more relevant than describing the aims of PPI such as democratizing health care etc. Please consider revising.

Is the study design appropriate for the research question?

Yes

Is the rationale for, and objectives of, the study clearly described?

Partly

Are sufficient details of the methods provided to allow replication by others?

Yes

Are the datasets clearly presented in a useable and accessible format?

Not applicable

Reviewer Expertise:

Quality improvement, patient safety, coproduction, organisational learning.

I confirm that I have read this submission and believe that I have an appropriate level of expertise to confirm that it is of an acceptable scientific standard, however I have significant reservations, as outlined above.

## References

[1] : Learning health systems using data to drive healthcare improvement and impact: a systematic review. *BMC Health Serv Res* .2021;21(1) : 10.1186/s12913-021-06215-8200 10.1186/s12913-021-06215-8 33663508 PMC7932903

[2] : Coproduction of healthcare service. *BMJ Qual Saf* .25(7) : 10.1136/bmjqs-2015-004315509-17 10.1136/bmjqs-2015-004315 26376674 PMC4941163

[3] : Complexity Science as a Frame for Understanding the Management and Delivery of High Quality and Safer Care.2021; 36315752
